# Neurobehavioural signatures in race car driving: a case study

**DOI:** 10.1038/s41598-020-68423-2

**Published:** 2020-07-14

**Authors:** Ines Rito Lima, Shlomi Haar, Lucas Di Grassi, A. Aldo Faisal

**Affiliations:** 1 Brain and Behaviour Lab: Dept. of Bioengineering, Imperial College London, London, UK; 2 Dept. of Computing, Imperial College London, London, UK; 3 Behaviour Analytics Lab, Data Science Institute, Imperial College London, London, UK; 4 UKRI Centre for Doctoral Training in AI for Healthcare, Imperial College London, London, UK; 5 MRC London Institute of Medical Sciences, London, UK; 6 RoboRace, London, UK

**Keywords:** Motor control, Sensorimotor processing

## Abstract

Recent technological developments in mobile brain and body imaging are enabling new frontiers of real-world neuroscience. Simultaneous recordings of body movement and brain activity from highly skilled individuals as they demonstrate their exceptional skills in real-world settings, can shed new light on the neurobehavioural structure of human expertise. Driving is a real-world skill which many of us acquire to different levels of expertise. Here we ran a case-study on a subject with the highest level of driving expertise—a Formula E Champion. We studied the driver’s neural and motor patterns while he drove a sports car on the “Top Gear” race track under extreme conditions (high speed, low visibility, low temperature, wet track). His brain activity, eye movements and hand/foot movements were recorded. Brain activity in the delta, alpha, and beta frequency bands showed causal relation to hand movements. We herein demonstrate the feasibility of using mobile brain and body imaging even in very extreme conditions (race car driving) to study the sensory inputs, motor outputs, and brain states which characterise complex human skills.

## Introduction

One of the hallmarks of being human is our unique ability to develop skills and expertise. While all animals develop skills like walking, running, fruit picking or hunting, we as humans can develop a much broader and more diverse set of skills. With practice, most of us can learn to play a musical instrument, play a sport, or do arts and crafts. Nevertheless, only some of us can reach the highest level of expertise. Unlike the widespread view that this is entirely driven by practice^1^, there is accumulating evidence that practice is not enough^2^, making individual musicians, artists, athletes, and craftspeople who take this expertise to new heights of particular research interest. Novel technology for mobile brain and body imaging now enables us to study neurobehaviour in real-world settings^3–5^. When carried out in natural environments, these measures can enable a meaningful understanding of human behaviour while performing real-life tasks. Studying the relation and inter-dependencies between brain activity and body movements of experts, while they perform their expert skills in real-world settings, can enable us to unpack this enigma.

In recent years there has been an accumulating body of literature studying the neural signals associated with expertise, particularly in sports^6^. EEG studies link expert performance to changes in EEG alpha and beta rhythms. However, most of these studies are using lab-based tasks, and therefore their findings have had little impact on sports professionals^6^ (for example, the Go/No-Go task was used to study baseball expertise^7^). While their findings showed that experts perform better and have more robust EEG inhibition responses, which can tell us something about their skill, it is far from true expertise. Other studies addressed expertise in a real task in a trial-by-trial design. For example, expert rifle shooters exhibited longer quiet eye period before shooting and showed an increased asymmetry in alpha and beta power (increase in left-hemisphere and decrease in right-hemisphere) during the preparatory period^8^. Also, expert golf players show a more significant reduction in EEG theta, high-alpha, and beta power during action preparation^9^. Measuring and interpreting the neurobehaviour of expertise during the continuous performance of a real-world task is the next challenge. Here we present a case study in real-world neuroscience of expertise, measuring the brain activity and body movement of a professional race car driver (Formula E Champion) while driving under extreme conditions (high speed, low visibility and road slipperiness). Driving is a skill that most of us acquire and use on daily life. Previous literature on driving mostly addressed it as such and thus focused on evaluating brain signals, eye gaze, or body movements, in order to measure the state of attention^10,11^, fatigue^12–14^ and drowsiness^15,16^ of the driver, which are significant causes of road accidents. Here, we use driving as an exciting test-bed to demonstrate the feasibility of studying real-world expertise under extreme conditions in-the-wild. We address the driver’s natural perceptual input (vision), motor output (eyes and limbs movements) and brain activity comprehensively, in an attempt to assess the neuromotor behaviour responses under challenging driving conditions, which are hallmarks of expertise in race driving. As in any case study, this work is limited in key components of scientific inquiry such as comparison and generalisation. Further work is required to determine the specificity of the reported neural signatures to expertise and to distinguish it from non-experts. However, it highlights a unique neurobehavioral characteristic that could be tested for generalisation in future studies. This study, in essence, is a proof of concept, showing the feasibility of real-world neuroscience in-the-wild, even in extreme conditions. The second objective of this study was to characterise the neuromotor behaviour of a highly skilled expert in a high skill task, performed in real-life conditions, outside a laboratory or simulator, and thus creating a reference point for future real-world and simulator driving studies with experts and non-expert drivers. A better understanding of expert drivers’ neural and motor interdependencies while facing driving challenges can also potentially foster the development of technologies to prevent critical conditions and improve driving safety, as well as safety procedures for autonomous and semi-autonomous cars.

## Methods

### Experimental setup

The experiment took place at the Dunsfold Aerodrome (Surrey, UK), commonly known as the Top Gear race track. The driver (co-author) was Formula E champion, Lucas Di Grassi (Audi Sport ABT Schaeffler team), with over 15 years of professional racing experience, which include karting, Formula 3, Formula One and Formula E racing. The participant, LDG, is an author on this paper and gave informed consent to participate in the experiment and to publish the information and images in an online open-access publication. While all methods used in the study were approved by the Imperial College Research Ethics Committee and performed in accordance with the declaration of Helsinki, this study was a self-experimentation by an author^17^. The test drive was prescheduled for video production purposes by the racing team, who race in these conditions frequently. It enabled us this unique scientific observation of motor expertise in the wild. Although unlikely to be needed, emergency response units were present. A promo video of the film by Averner Films^18^ is accessible here: https://vimeo.com/248167533.

**Figure 1:**
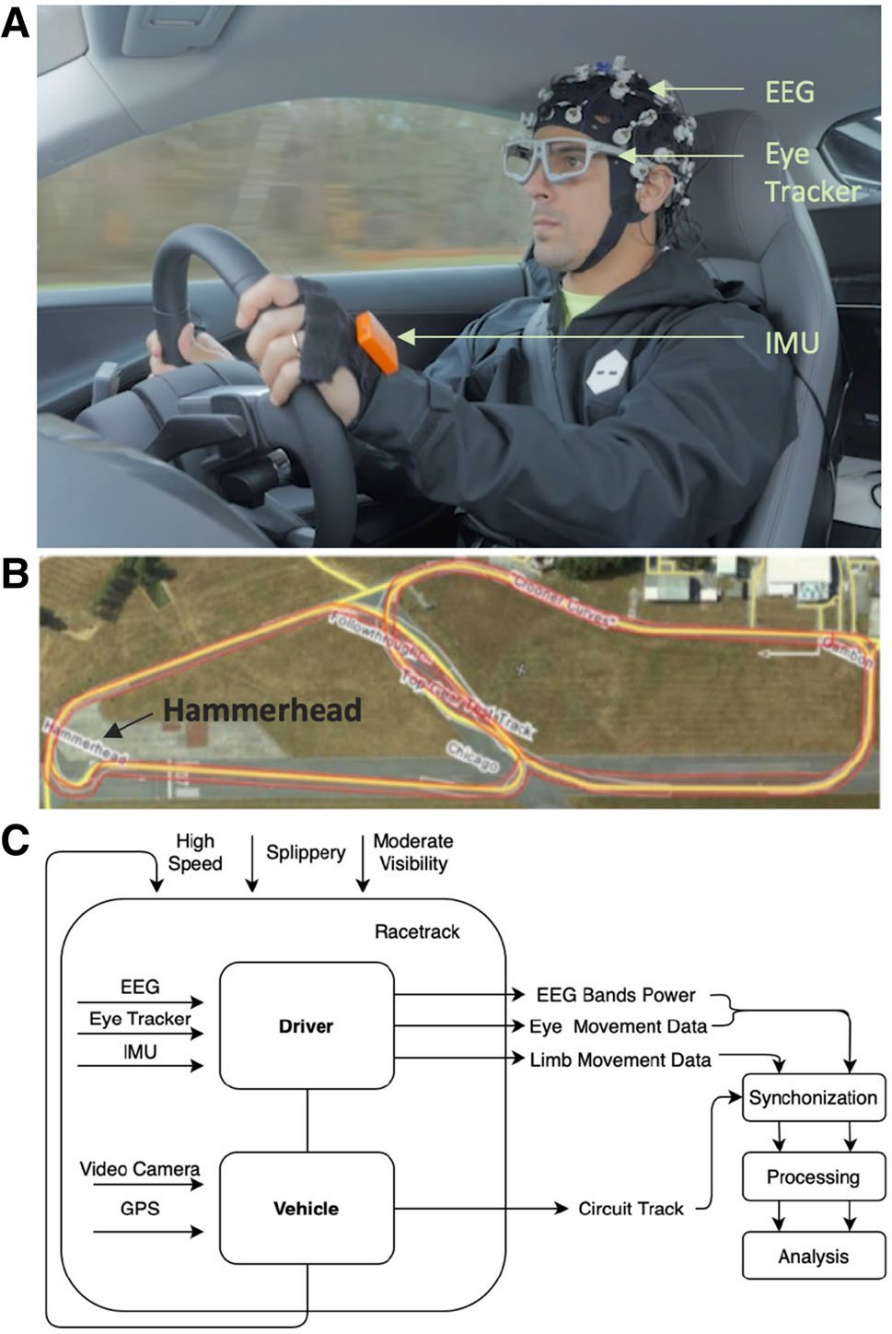
Experimental setup involving expert driver (LDG): (**A**) involved equipment (EEG, eye tracking and body motion tracking systems) and their placement on the driver; (**B**) Top Gear racetrack and the highlighted race critical curve, the *Hammerhead*; (**C**) architecture of our data collection system with driver/car/road parameters inputs and data flows.

The driver was equipped with: a 32-channels wireless EEG system (LiveAmp, Brain Products GmbH, Germany) with dry electrodes (actiCAP Xpress Twist, Brain Products GmbH, Germany); binocular eye-tracking glasses (SMI ETG 2W A, SensoMotoric Instruments, Germany); and four inertial measurement units (IMUs) on his hands and feet (MTw Awinda, Xsens Technologies BV, The Netherlands); shown in Fig. 1A. The car was equipped with a GPS and a camera recording the inside of the car. The car driver assistance systems were turned off. The full architecture of the experimental setup is presented in Fig. 1C.

The Top Gear race track has specific curve types that subjugate drivers’ to different driving challenges. In particular, in the south-west of the track (left side of of the image in Fig. 1B) is the *Hammerhead* curve. As the name suggests, it is a hammerhead-shaped tricky curve, designed to test cars and drivers’ skill for it is technically challenging^19^. This is considered the critical curve of the track, being used in this work to assess the driver’s performance in challenging scenarios. This extreme curve was selected to show highlight features of the driver’s neuromotor behaviour. The car was driven with a mean driving speed of 120 Km/h and a top speed of 178 Km/h on a track 2.82 Km long with 12 curves. The experiment consisted of 6 sets of 2 to 3 laps each, stopping after every set for system check-up and recalibration.

### Data processing

All data were pre-processed using custom software written in MatLab (R2017a, The MathWorks, Inc., MA, USA). During the recording in the car, all data streams were synchronised using the computer’s real-time clock (RTC). We verified the synchronisation offline and corrected for any drifts and offsets by computing temporal cross-correlations. Since the car acceleration had an equal effect on the accelerometers of the IMUs on the limbs and the built-in IMU in the GPS and the EEG headset, timestamps were synchronised across all data these streams using cross-correlation between the accelerations recorded by all systems and minor offsets between timestamps were corrected for each six sets. The gaze data was synced to the motion capture sensors (Xsens) using the video from the eye tracker’s egocentric camera, in which the driver’s hands are clearly visible. During the race, the driver was repeatedly in states of almost complete stillness (while driving on a straight path) which were followed by rapid hand movements (as the car started to slip on the road or drove into a sharp curve). We automatically detected these extreme peaks in hand acceleration and then looked at the video frames around those peaks and corrected for the minor offsets (of at most 33 msecs, based on 30 fps of the egocentric camera) between timestamps. Then data streams were segmented to the laps, and laps with missing data in either stream were removed.

#### Car movement data

The car GPS was obtained by the placement of an iPhone inside the car. This system’s acceleration and rotation recordings were resampled to a stable 100 Hz sampling rate, for timestamp synchronisation and for cleaning the body data. The data was filtered resorting to a zero phase-lag fourth-order Butterworth filter with 10 Hz cut off frequency^20,21^.

#### Body movement data

Body data was collected by 4 Xsens MTw Awinda IMUs placed on hands and feet. These sensors recorded rotation and acceleration. The data collected has a stable sampling rate of 100 Hz. Since the driver was in a moving car, the motion captured by the motion capture system was the combination of the car movement and the driver’s limb movements inside the car. Linear regression was implemented in order to clean the car movement (based on the GPS recordings) from the motion tracking. The regression’s residuals capture the movement of the limbs that do not fit the car movement, for rotation and acceleration data separately. The data was then filtered with resorting to a zero phase-lag fourth-order Butterworth filter with 10 Hz cut off frequency^20,21^.

#### Gaze data

The eye gaze was acquired with binocular SMI ETG 2W A eye-tracking glasses which use infrared light and a camera to track the position of the pupil. Using the pupil centre and the corneal reflection (CR) creates a vector in the image that can be mapped to coordinates^22,23^. The use of eye-tracking glasses simplify the analysis of gaze-targets inside a free moving head in a freely driving car, as those are not calibrated to a fixed physical coordinated system but only to the device’s built-in egocentric camera that captures the visual scene that the subject sees. The glasses and the camera move with the head and thus no correction for head movements is needed. The egocentric camera captured the scene view and therefore enabled the reconstruction of the frame-by-frame gaze point in the real-world scene (e.g. visual relations of gaze-target to the side of the road). The gaze data was collected at 120 Hz and included multiple standardised eye measurements, such as pupil size, eye position, point of regard, and gaze vector. When measuring gaze behaviour in-the-wild, fixation and saccades become an under-specified concept^24^. For example, a free-moving head tracking a location on a moving road involves smooth eye movements and saccades during fixation, which are considered mutually exclusive in fixed head settings. Therefore, conventional definitions of these types of eye-movements and ways of analysis do not apply. Thus, our analysis focused on the point of regard, obtained from the RMS between the point of regard binocular measured in X and Y axis; the gaze vector, computed for the right and left eyes, separately, through the RMS of XYZ; and the change in gaze vector, calculated using two consecutive measurements. These measures were resampled to 100 Hz, and linear interpolation was applied to account for some missing data points in the gaze recording.

#### EEG data

The brain activity was recorded using a 32 channels EEG cap with dry electrodes (displayed accordingly to the 10-20 system), sampled at 250 Hz stable sampling rate. EEG data was analysed with EEGLAB toolbox (https://sccn.ucsd.edu/eeglab;^25^) . The processing steps included (i) high pass filtering with a cutoff frequency of 1 Hz^26–28^; (ii) line noise removal in the selected frequencies of 60 Hz and 120 Hz^26^; (iii) bad channels removal for those with less than 60% correlation to its own reconstruction based on its neighbour channels^26^; (iv) re-referencing the EEG dataset to the average (of all channels) to minimise the impact of a channel with bad contact in the variance of the entire dataset^26^; (v) Independent Component Analysis (ICA) in order to separate signal sources^29–31^; (vi) Artefacts removal using *runica, infomax* ICA algorithm from EEGLAB, for identification and removal of head and eye movements and blinking artefacts. The algorithm searches for the maximal statistical independence between sources. Artefacts sources were identified based on a predefined set of criteria for scalp topographies and spectrum analysis (e.g. source location by the ears and power spectrum with high and spiky power in high frequencies indicates movement artefacts, source location between the eyes suggest eye blink artefacts, source location in the eye indicates lateral eye movement artefacts)^25^. EEG data was then transformed in the time-frequency domain to power in decibels (dB) in 100 Hz (to match the other data streams), in *delta* ($\delta$) 0.5 to 4 Hz; *theta* ($\theta$) 4 to 8 Hz; *alpha* ($\alpha$) 8 to 12 Hz; and *beta* ($\beta$) 12 to 30 Hz frequency bands. The transformation was applied separately to the individual IC located over the left motor cortex and to the mean brain activity, averaged across the cleaned ICs.

## Results

The results section of this case-study paper were written to characterise the neuromotor behaviour of a professional driver while driving in extreme condition, which can be used as a reference point for future driving studies. Initial analysis was aimed to understand the interdependencies between the different neurobehavioural data streams and their level of complexity. The analysis then focused on specific driving events, such as response to challenging conditions (skidding, curves and straights), in order to assess if there is a distinguishable behaviour upon those moments. Lastly, we addressed the causality across different data streams.

### Data characteristics

**Figure 2:**
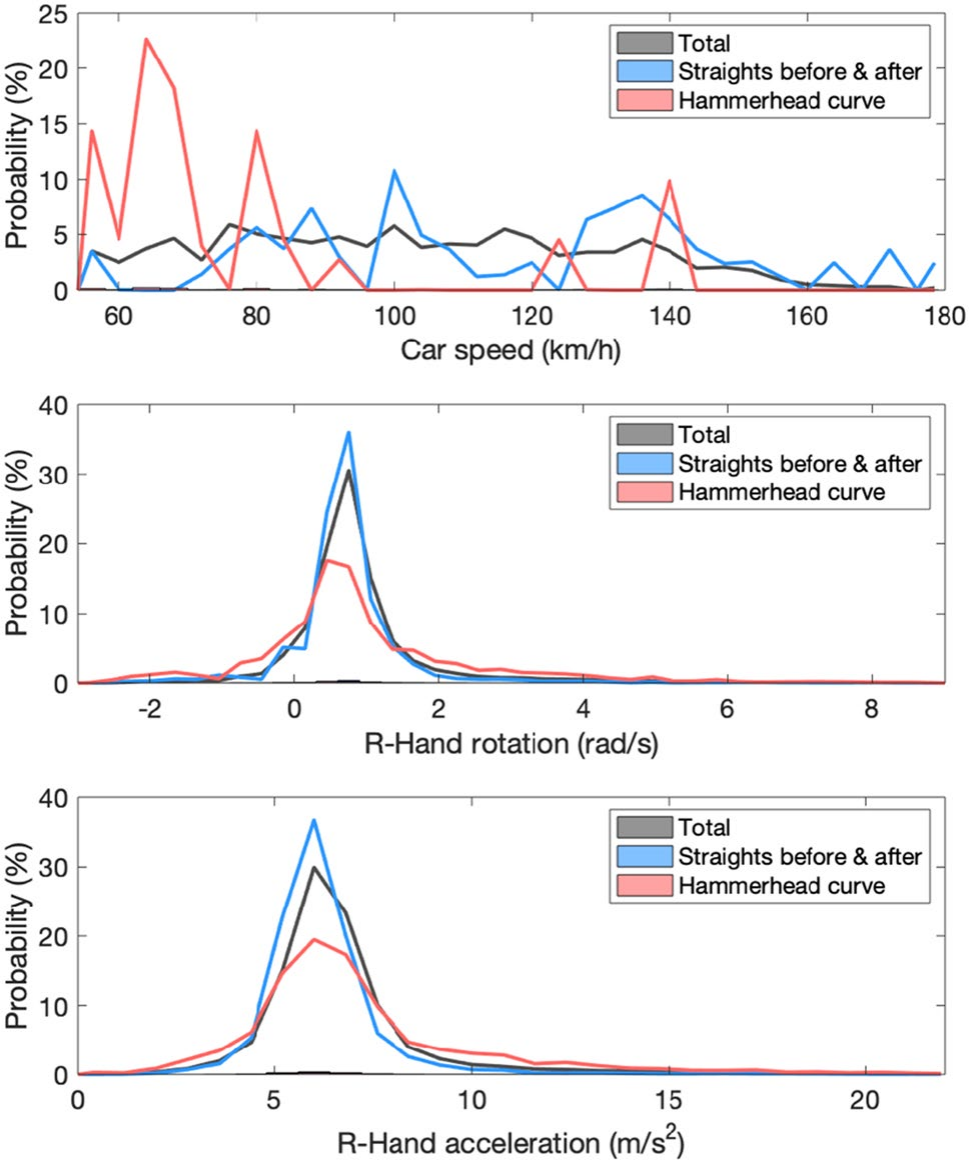
Histogram evolution analysis detail the extreme driving scenario of this experiment. (top) car speed with an average of 120Km/h, critical curve speed average of 78 Km/h and straight speed average of 130 Km/h, all above conventional driving speed limits; (middle) right-hand gyroscope with an average value of 0.9 rad/s whereas for intense driving style (critical curve and skidding moments) the average values are of 1 and 3 rad/s, superior to normal movement expected from literature; and (bottom) right-hand accelerometer data showing absolute acceleration with similar results distribution as gyroscope data. Data regarding straights corresponds to 25.5% of the entire data set and the Hammerhead curve to 11.3%.

The distributions of the car speed, the right-hand rotation, and the right-hand accelerations were assessed in the entire track, the straight segments before and after the hammerhead curve, and the hammerhead curve itself (Fig. 2). The average speed throughout the experiment was 120Km/h. As expected, in the curves speed was relatively low (between 54 and 82 Km/h), while in straight segments speed was much higher with broader distribution, as the car decelerated towards a curve and accelerated after the curve. The number of frames considered hammerhead critical curve was 11.3% of the total recording, and straights corresponded to 25.5%.

Since the hand movements were highly correlated here, we show only the right hand. Both gyroscope and accelerometer distributions present similar tendencies, with a narrow distribution during the straight segments and a slightly wider one in curves. The result considering the whole data set lies in-between. The gyroscope values for the abrupt responses (skidding) have a mean of 3rad/s, considerably superior to the 1rad/s found in the literature for normal forearm movement^32^, which is expected considering the intense car handling. Data considered as abrupt responses corresponds to 6% of the dataset.

**Figure 3:**
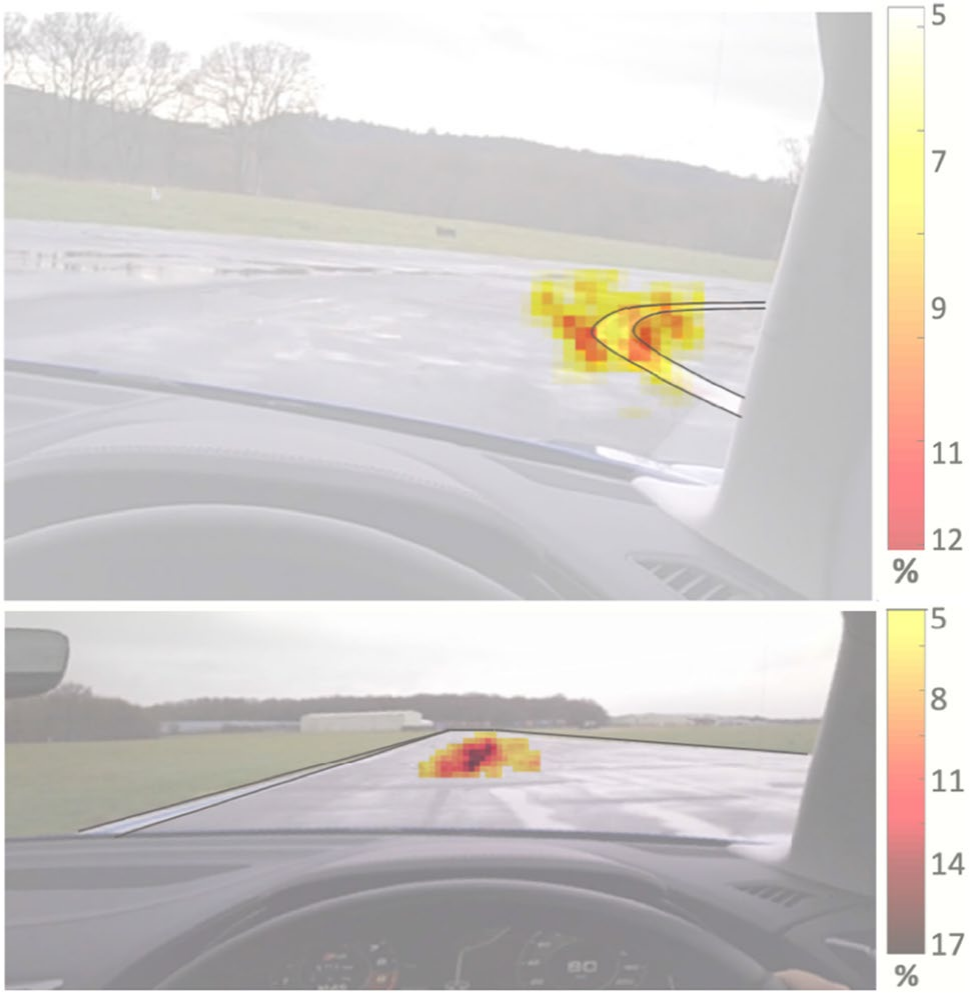
Heat maps of eye gaze using data recorded during the *Hammerhead* critical curve (top), highlighting the driver’s tendency of tracking the tangent curve; and the straight segments before and after the curve (bottom), where the driver’s gaze focus on the horizon.

Eye gaze data showed a strong tendency of tracking the tangent point of the curve, as illustrated in Fig. 3 (top), and reported for non-expert drivers^33^. In the top figure the eye gaze search for the tangent of the curve can be seen (internal and external), marked in white on the road, where it remains throughout the entire curve. During straight segments, the eye gaze focuses straight ahead, with a stable distance in the horizon, with minor saccadic deviations, as illustrated in Fig. 3 (bottom). The heat map was built using data recorded during the critical curve (top) or the straights before and after that curve (bottom). The gaze point position from the geocentric view was annotated at a ten frames cadence in an overlapping position matrix. The heat map was built resorting to the percentual annotations occurrence in this matrix.

### Global dataset assessment

**Figure 4:**
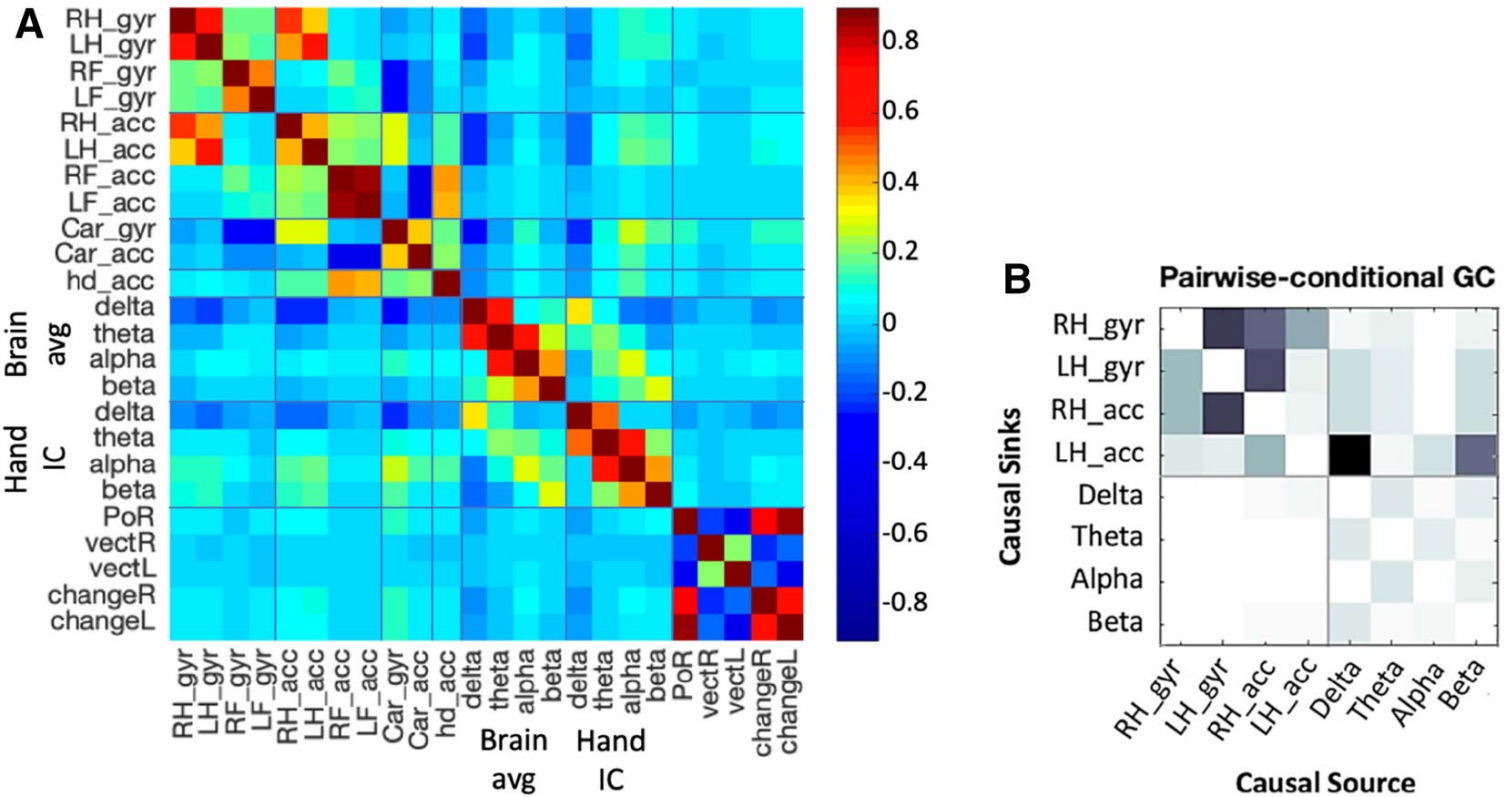
Global assessments: (**A**) Instantaneous correlation matrix between all data groups: gyroscope rotation (gyr) and acceleration (acc) of the right and left hands (RH,LH), the right and left feet (RF,LF), the car (Car), and the head (hd); the band power in the delta, theta, alpha, and beta bands across the brain (Brain avg) and in the left motor cortex (Hand IC); eye gaze point of regard (PoR), the gaze vectors (vect) and the change in gaze vector (change) for the right (R) and the left (L) eye; (**B**) Granger causality pairwise results considering the data groups separated by grey lines. Darker colour is associated with more robust causality, for which brain data is more cause of hand movements than the other way around. A significance of p=0.05 and Bonferroni correction supported these conclusions.

For an overall understanding of the interdependencies between the neurobehavioral data streams, a correlation matrix was computed (Fig. 4A). The variables considered were: the RMS of the acceleration and the rotation over the four limbs, the car, and the head; EEG band power for the mean brain activity, and the right hand IC; and eye movement data including the point of regard, the gaze vectors, and the change in gaze vector for both eyes. The right and the left hands are strongly correlated, as expected since the driver had both hands on the steering wheel. There are other correlations within domains (e.g. neighbouring band powers are correlated), and also weaker, but statistically significant, correlations between domains. Most importantly, between the EEG band powers and the hand movements. We found a positive correlation for the hands’ acceleration and rotation with the alpha and beta power in the right hand IC, and a negative correlation with the delta power (P < 0.001). Cross-correlation shows that while the delta band is synced with the movement, the neural signal in the alpha and beta band precedes the movement by  100 ms.

#### Granger causality

To address the sequence of causality between variables, we used Granger causality^34^. Granger causality is based on the idea that causes precede and help predict their effects. This technique tries to identify direct interactions between time series, in both time and frequency domains. Granger causality was calculated using EEGLAB toolbox MVGC multivariate Granger causality (mvgc_v1.0)^35,36^. The toolbox uses autoregressive vector modelling to find linear interdependencies between time series based on their past values. The Granger causality analysis was conducted on the hands’ acceleration and gyroscope rotation data, and EEG bands power from the IC of the right hand. The results are presented in Fig. 4B as a pairwise matrix of causality between causal sources to causal sinks (darker indicates more robust causality) for statistically significant causality links (p<0.05 after Bonferroni correction). We found one-directional causality where the EEG bands power are causing hand movements, specially *delta* and *beta*.

### Specific driving related events

**Figure 5:**
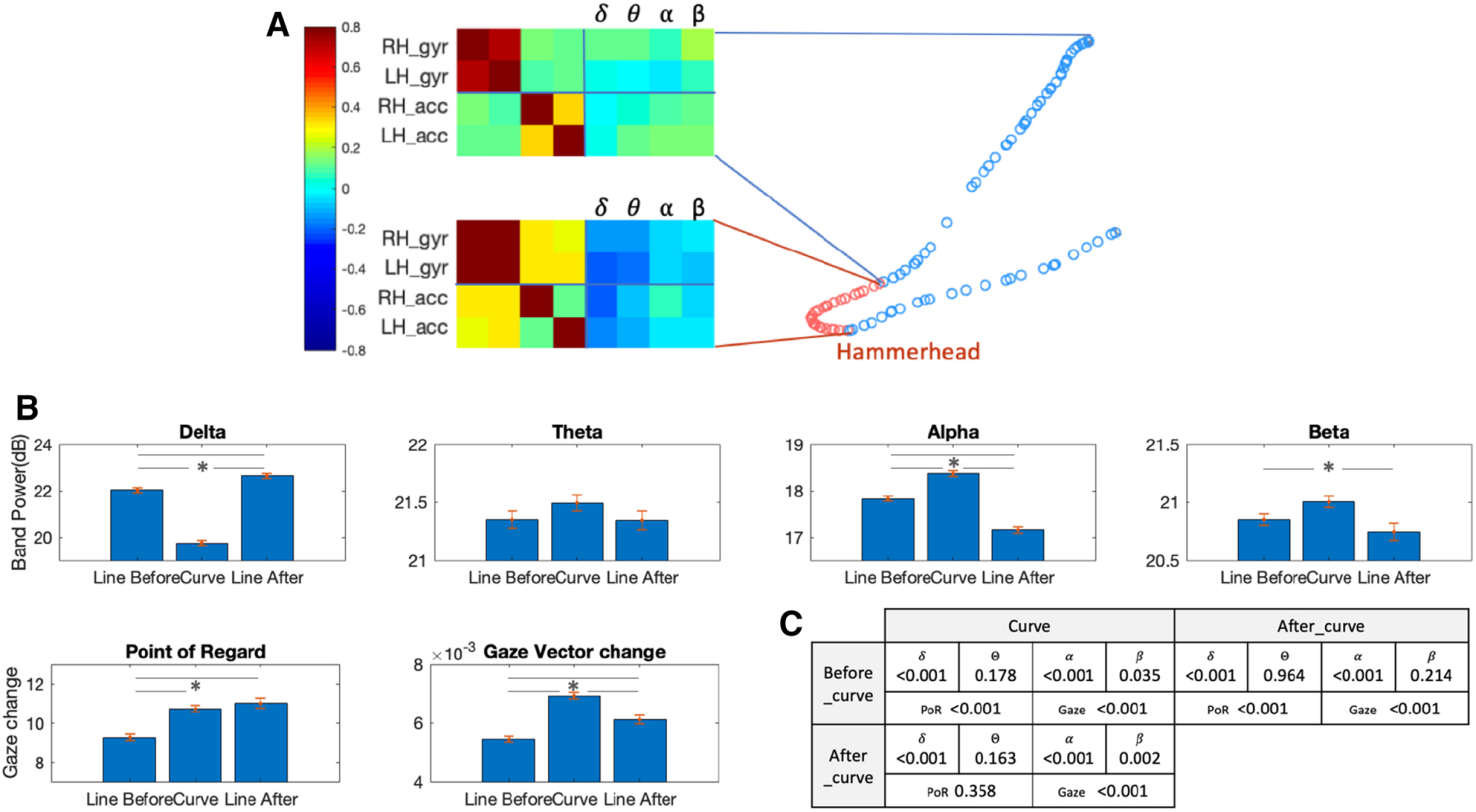
(**A**) Identification of the straights (blue) and curve (red) periods, with a fragment of the correlation matrix obtained for each period; (**B**) statistical analysis on the mean and SEM for curves and straights on bands power and eye data. Despite lower correlation values, the brain shows structured correlation to the hand when performing the curve, with *delta* and *theta* anticorrelation and *alpha* and *beta* positive correlations, instead of a generalised correlation to the body. Statistical significance between before, during and after the critical curve was found for *delta*,*alpha*,*beta*, point of regard and change in gaze vector. (**C**) shows the p-values obtained with t-test.

Here we considered a comparison between the neurobehavioral signature of the challenging Hammerhead curve and the straight segments leading to it and out of it. Based on the GPS data and the egocentric videos, we annotated the segments of the critical *Hammerhead* curve in the track and the straight paths leading to it and out of it. Correlation matrix from hands IMUs and EEG data streams for these segments shows differences in the correlation structure between the two types of segments (Fig. 5A). While in the straight segments, there was no correlation between the hands’ rotation and acceleration to the EEG power, in the curves, there was a negative correlation between these brain and body measurements. Statistical analysis of the differences between the segments shows a decrease in *delta* power and an increase in *alpha* and *beta* during the curve (Fig. 5B, C). The data from the segments before and after the curve also showed differences where *delta* power was lower before and higher after the curve, while *alpha* power showed the opposite trend. Eye gaze vector also changed more during the curves.

## Discussion

This work is an attempt to assess neurobehavioural signatures of real-world motor behaviour in-the-wild. We demonstrate the feasibility of simultaneously measuring brain activity, eye and body movement behaviours in-the-wild under extreme conditions (race track driving), assessing neurobehavioural interdependencies and inferring causal relations. First, we have demonstrated that body movements, eye movements, and wireless EEG data can be collected in very extreme conditions in-the-wild. Second, we have demonstrated that these measurements are meaningful not only from a purely descriptive experience but also by showing how they are predictive of each other, how they are related to car performance and race track location. And third, we used this data to characterise the neuromotor behaviour of an expert while driving in extreme conditions, which can be used as a reference point for future driving studies. This proof of “possibility” will act as a foundation to enable further research in real-world settings.

Our results show changes in the EEG power and the gaze characteristic during sharp curves, where the control of the car is most challenging. While many previous studies found correlations between hand movements and brain activity in lab-based repeated trials tasks^37^, here we show such correlations in continuous movement in-the-wild. Moreover, in a controlled lab experiment, there is a clear trial order where the timing of stimuli appearance, go-cue, etc. are well defined. Accordingly, the direction of the causality (if it exists) is clear -neural activity after a stimulus can be caused by it but cannot cause it. At the same time, neural activity before movement can cause the movement but cannot be caused by it. In-the-wild, causal relationships may reverse or be bi-directional. Here we show not only the correlation but the causality from the brain activity to the body movement in an unconstrained setting. Interestingly, the EEG power changes are in line with previous results on general creative solution finding and interventions^38^.

Comparing the driver’s neurobehaviour between the sharp curves and the straight segments before and after, enabled us to assess world-championship-level skilled responses while facing extreme driving conditions. Our results suggest seeming differences in the EEG power, point of regard, and gaze change vector, between the different segments: before-during-after sharp curves. The difference between the driving segments means that we can detect neurobehavioural differences between more and less demanding segments of the drive from on-going in-the-wild EEG and gaze recordings. It suggests possible neurobehavioural matrices for task demand that would presumably be different in expertise. During the curves, the driver showed a power increase in the alpha and beta bands and a decrease in the delta band. The increase in alpha is potentially a signature of the increased creativity demand in these segments^38,39^. The alpha and beta power increase we observed are also in line with previous work showing an increase in left-hemisphere alpha and beta power of expert rifle shooters during the preparatory pre-shot period^8^.

Neurobehavioural data collection in-the-wild is subject to more noise sources and interference than standard data collection in-the-lab. This concern is specifically worrying for the EEG signal, which is always contaminated by noise, and any EEG recording during movement is subject to movement and muscle artefacts. Thus, we find our cross-correlation and Granger causality results very encouraging, as those suggest the EEG activity precedes the movement and predicted it and not the other way around. If the EEG results were simply movement artefacts, we would have expected to see the opposite causality - the movement would precede and predict the EEG movement artefact. Thus, since the EEG activity precedes the movement, we believe the EEG results cannot be rejected as noise artefacts.

The driver’s gaze during curves followed the tangent point of the curve, as suggested in the classic paper by Land and Lee^33^. During the straight segments, his gaze was entirely focused on the centre of the road which led to the more stability in straight segments relative to curves, though during both segments type the driver’s gaze is exceptionally stable, as illustrated in Fig. 3.

Being able to collect real-world data which capture a significant portion of the sensory input to the brain (visual scene and locus of attention), the motor output of the brain (hand, head and arm movements during driving) as well as the state of the brain (EEG signals), is a further realisation of our human ethomics approach. This is not only insightful for understanding the brain and its behaviour, but also for devising artificial intelligence to improve driving safety for autonomous and semi-autonomous cars. In recent work^40^, we demonstrated how human drivers in a virtual reality driving simulator generated gaze behaviour that we used to train a deep convolutional neural network to predict where a human driver was looking moment-by-moment. This human-like visual attention model enabled us to mask “irrelevant” visual information (where the human driver was not looking) and train a self-driving car algorithm to drive successfully. Crucially, our human-attention based AI system learned to drive much faster and with a higher end-of-learning performance than AI systems that had no knowledge of human visual attention. The work we present here takes this *en passant* human-annotation of skilled behaviour to the next level, by collecting real-world data of rich input and output characteristics of the brain. Similarly to the way we used drivers’ gaze in a driving simulator to train a self-driving car algorithm in that simulator, we can use rich neurobehavioural data from an expert driver in extreme conditions to train a real self-driving car algorithm to response successfully in extreme conditions. Likewise, on the side of control systems, we showed for example how using ethomic data obtained from natural tasks (movement data^41^, electrophysiological data^42^, decision making data^43^) can be harnessed to boost AI system performance. The neurobehavioural approach demonstrated here suggests how we can succeed in future to close-the-loop between person and vehicle.

In summary, we demonstrated the feasibility of studying the neurobehavioral signatures of real-world expertise in-the-wild. We showed evidence of specific brain activity and gaze patterns during driving in extreme conditions, in which, presumably, the expertise of the driver makes a crucial difference. Future work is required to generalise these findings from this single case study.
